# Two-way plant mediated interactions between root-associated microbes and insects: from ecology to mechanisms

**DOI:** 10.3389/fpls.2013.00414

**Published:** 2013-10-23

**Authors:** Nurmi Pangesti, Ana Pineda, Corné M. J. Pieterse, Marcel Dicke, Joop J. A. van Loon

**Affiliations:** ^1^Laboratory of Entomology, Wageningen UniversityWageningen, Netherlands; ^2^Plant-Microbe Interactions, Institute of Environmental Biology, Utrecht UniversityUtrecht, Netherlands

**Keywords:** insect herbivores, induced systemic resistance, mycorrhizae, plant growth promotion, phytohormones, parasitoids, rhizobacteria, rhizobia

## Abstract

Plants are members of complex communities and function as a link between above- and below-ground organisms. Associations between plants and soil-borne microbes commonly occur and have often been found beneficial for plant fitness. Root-associated microbes may trigger physiological changes in the host plant that influence interactions between plants and aboveground insects at several trophic levels. Aboveground, plants are under continuous attack by insect herbivores and mount multiple responses that also have systemic effects on belowground microbes. Until recently, both ecological and mechanistic studies have mostly focused on exploring these below- and above-ground interactions using simplified systems involving both single microbe and herbivore species, which is far from the naturally occurring interactions. Increasing the complexity of the systems studied is required to increase our understanding of microbe–plant–insect interactions and to gain more benefit from the use of non-pathogenic microbes in agriculture. In this review, we explore how colonization by either single non-pathogenic microbe species or a community of such microbes belowground affects plant growth and defense and how this affects the interactions of plants with aboveground insects at different trophic levels. Moreover, we review how plant responses to foliar herbivory by insects belonging to different feeding guilds affect interactions of plants with non-pathogenic soil-borne microbes. The role of phytohormones in coordinating plant growth, plant defenses against foliar herbivores while simultaneously establishing associations with non-pathogenic soil microbes is discussed.

## INTRODUCTION

Plants are members of complex communities and function as a link between above- and below-ground communities that consist of microbes, insects, and other vertebrate and invertebrate animals (Bezemer and van Dam, 2005; Dicke and Baldwin, 2010). In addition to a multitude of direct interactions between these different community members, indirect interactions occur via shared host plants (Ohgushi, 2005; Kaplan and Denno, 2007; Gehring and Bennett, 2009; Pineda et al., 2010). To survive, plants need to optimally allocate resources to growth and defense (Herms and Mattson, 1992). For instance, in the presence of plant pathogens or insect herbivores, plants will allocate resources to the synthesis of defense compounds and as a consequence plant growth will decrease. Remarkably, plants form associations with non-pathogenic root-associated microbes such as mycorrhizae, rhizobia, and rhizobacteria that can promote plant growth by increasing their access to soil minerals (Mendes et al., 2011; Berendsen et al., 2012; Bulgarelli et al., 2013). Moreover, several species of non-pathogenic root-inhabiting microbes can trigger physiological changes and induction of defenses in the host plant that have systemic effects on aboveground insect communities involving organisms at several trophic levels (Leitner et al., 2010; Pineda et al., 2010, Pineda et al., 2013; Katayama et al., 2011b). Most studies in this area, however, mainly address plant interactions with single species of non-pathogenic microbes. In recent years, the root microbiome as a whole has appeared crucial for many aspects of plant development and immunity (Hol et al., 2010; Mendes et al., 2011; Partida-Martinez and Heil, 2011; Berendsen et al., 2012; Martinuz et al., 2012). Therefore, a shift should be made from studying single microbial species to investigating the community of root inhabiting microbes and its effects on plant–insect interactions.

Aboveground, plants are under continuous attack by various organisms such as insects and pathogens and mount multiple responses that have systemic effects on belowground microbes. Insect leaf chewing, for instance, leads to reduced leaf area and, therefore, reduced photosynthetic potential which may affect allocation of resources to the roots and the level of root exudation (Gehring and Bennett, 2009). Furthermore, induced plant defenses against plant pathogens or insect herbivores can alter concentrations of secondary metabolites in the shoots and roots that influence plant interactions with non-pathogenic soil microbes. During the past few years, evidence has accumulated that plants have a sophisticated defense mechanism by actively recruiting non-pathogenic root-associated microbes following attack by pathogens or insects (Rudrappa et al., 2008; Lakshmanan et al., 2012; Lee et al., 2012b). By regulating its root secretion in the form of carbon-rich exudates, plants can actually shape the root microbiome by affecting microbial diversity, density, and activity (Barea et al., 2005; Dennis et al., 2010). More recently, significant progress has been made in understanding signaling pathways and molecules involved in recruitment of specific groups of microbes following foliar herbivory and defense activation (de Roman et al., 2011; Yang et al., 2011b; Yi et al., 2011; Doornbos et al., 2012; Lakshmanan et al., 2012; Landgraf et al., 2012; Lee et al., 2012b; Neal et al., 2012).

As sessile organisms, plants rely on a range of chemical compounds to repel enemies and attract mutualistic organisms above- and below-ground (Rasmann et al., 2005; Dicke and Baldwin, 2010). The phytohormones jasmonic acid (JA) and salicylic acid (SA) function as major players in coordinating the complex signaling pathways involved in these multitrophic interactions (Robert-Seilaniantz et al., 2011; Pieterse et al., 2012). Other plant hormones such as ethylene (ET), abscisic acid (ABA), cytokinin (CK), gibberellin (GA), and auxin function as modulators of the hormone signaling backbone (Robert-Seilaniantz et al., 2011; Meldau et al., 2012; Pieterse et al., 2012; Giron et al., 2013). The underlying molecular pathways mediating plant–insect and plant–microbe interactions are interconnected. Induction of the JA- and SA-signaling pathways depends on the mode of feeding of the herbivorous insect species (De Vos et al., 2005; Wu and Baldwin, 2010; Erb et al., 2012; Thaler et al., 2012; Soler et al., 2013). In interactions between non-pathogenic rhizosphere microbes and plants, the phytohormones JA, SA, and ET regulate symbiosis and mediate induced systemic resistance (ISR) elicited by several groups of non-pathogenic microbes (De Vleesschauwer et al., 2009; Zamioudis and Pieterse, 2012). Moreover, recent experimental evidence has started to unveil the signaling pathways induced by root-associated microbes to stimulate plant growth. Here, we will review the role of these signaling pathways and their crosstalk in shaping microbe–plant–insect interactions. We have previously proposed that different groups of non-pathogenic microbes have similar plant-mediated effects on insect herbivores aboveground (Pineda et al., 2010). Since then, the field of non-pathogenic microbe–plant–insect interactions has made significant advances. Here, we review those recent findings and outline future perspectives.

## FROM EFFECTS OF MICROBES ON SINGLE HERBIVORE SPECIES TO EFFECTS ON INSECT COMMUNITIES

The field of microbe–plant–insect interactions has mainly addressed how a certain microbe affects single herbivore species. In nature, however, plants are sequentially or simultaneously attacked by multiple herbivores, that in turn are attacked by parasitoids and predators. It is therefore not surprising that effects of non-pathogenic microbes on a specific herbivore species will depend on how such an herbivore is interacting with the community of herbivorous insects. For instance, colonization of four grass species by the mycorrhizal fungus *Rhizophagus irregularis *(formerly known as *Glomus intraradices*) leads to a significant increase in performance of the generalist caterpillar *Spodoptera littoralis* as well as in aboveground plant biomass (Kempel et al., 2010). Interestingly, if the plants had been previously attacked by the same herbivore species, mycorrhization reduces the performance of a subsequent attacker as well as shoot biomass. The authors suggested that in herbivore-induced plants, mycorrhizal colonization mediates a shift of resource allocation from promoting plant growth to inducing resistance against insects. Whether plant signaling pathways are involved in this shift of resource allocation remains to be elucidated. In response to attack by multiple insect herbivores, plants activate different hormone signaling pathways depending on feeding characteristics of the insects (De Vos et al., 2005; Li et al., 2006). Recent studies show that induction of JA-dependent defenses against leaf chewers can be attenuated by previous infestation of phloem feeders such as aphids and whiteflies that activate the SA signaling pathway resulting in JA–SA antagonistic crosstalk mechanisms (Rodriguez-Saona et al., 2010; Soler et al., 2012; Zhang et al., 2013). How non-pathogenic microbes can modify the interaction between multiple herbivores is a question that has not been explored so far.

From a multitrophic perspective, during the past few years several studies have addressed the effects of below-ground non-pathogenic microbes on third-trophic-level organisms i.e., arthropod predators and parasitoids, via changes in the emission of herbivore-induced plant volatiles (HIPVs; Leitner et al., 2010; Hoffmann et al., 2011a,b,c; Katayama et al., 2011a; Schausberger et al., 2012; Ballhorn et al., 2013; Pineda et al., 2013). A set of studies with *Phaseolus vulgaris* bean plants showed that the mycorrhizal fungus *Glomus mosseae* resulted in reduction of spider-mite damage. In these studies mycorrhizae provided plants with a fitness benefit (i.e., increase of seed production) despite the increased performance of the herbivorous spider mite *Tetranychus urticae*, by enhancing the attraction and performance of predatory mites that feed on the spider mite (Hoffmann et al., 2011a,b). Increased emission of β-ocimene and β-caryophyllene in mycorrhizal-colonized bean plants was associated with the attractiveness to the predatory mite (Schausberger et al., 2012). However, root-associated microbes can also have negative plant-mediated effects on indirect plant defense. Colonization of *Arabidopsis thaliana* roots by *Pseudomonas fluorescens* modified HIPV emission after infestation by the generalist aphid *Myzus persicae *via JA-signaling and these changes reduced the attraction of the aphid parasitoid *Diaeretiella rapae *to the plants (Pineda et al., 2013). Thus, non-pathogenic root-associated microbes can have positive or negative effects on the attraction of organisms at the third trophic level. Which molecular mechanisms are underlying these contrasting effects remains to be elucidated and may explain why in some interactions positive and in others negative effects on indirect plant defense occur.

In addition to the effects on plant volatiles, several root-colonizing microbes can also produce volatiles themselves. These microbial volatiles have a role in plant growth promotion and ISR against pathogens (Choudhary et al., 2008; De Vleesschauwer et al., 2009; Lee et al., 2012a; Bulgarelli et al., 2013; Zamioudis et al., 2013). For instance, the short-chain volatile organic compound (VOC) 2,3-butanediol is produced by root-associated *Bacillus subtilis* GB03 and *Bacillus amyloliquefaciens* IN937a, and it can trigger ISR in *A. thaliana *against the pathogen *Erwinia carotovora* via the ET signaling pathway (Ryu et al., 2004). Interestingly, 2,3-butanediol is also known as insect attractant (Bengtsson et al., 2009; del Pilar Marquez-Villavicencio et al., 2011). Therefore, in addition to the indirect effects of microbes on herbivores via plant-mediated mechanisms, compounds produced by non-pathogenic root microbes could also have a direct effect on insect attraction. In this research topic, Kupferschmied et al., 2013 show insecticidal activity of some rhizobacteria-derived compounds. These direct effects of root-colonizing microbes on insect herbivores and their natural enemies need to be further assessed to gain a thorough understanding of their role in shaping plant-associated communities.

## MOVING FROM EFFECTS OF SINGLE MICROBE SPECIES TO THE COMMUNITY OF ROOT-ASSOCIATED MICROBES

The microbe–plant interaction can start as early as the seed formation, e.g., many endophytes are transmitted to the seeds via the parental plant (Gundel et al., 2011). Once the seed germinates in the soil, colonization of plant roots by multiple microbial species starts (Partida-Martinez and Heil, 2011). The majority of plant-associated microbes resides in the thin soil layer that is influenced by plant roots called rhizosphere, a dynamic niche in the soil that is strongly affected by the release of root exudates (Barea et al., 2005; Lundberg et al., 2012). The microbial community associated with plant roots, the so-called rhizosphere microbiome, has an important role in plant health and survival (Bakker et al., 2013; Mendes et al., 2013). The effects of the rhizosphere microbiome on ISR have mainly been studied for plant–pathogen interactions (Mendes et al., 2011), although mechanistic studies on the effects of the microbiome on ISR against herbivores have been initiated (Badri et al., 2013). A study by Hol et al., 2010 demonstrated the importance of evaluating the soil microbiome as a whole when studying microbe–plant–insect interactions. This study showed that the reduction of in particular microbes occurring at low abundance resulted in an increased aphid body size, as well as an increase in the biomass of *Beta vulgaris* and *Brassica oleracea*. However, until now a more frequently used approach to increase the complexity in studies of microbe–plant–insect interactions has been the use of a combination of several microbial strains. To properly determine the effect of these mixtures, it is required to also evaluate the effect of the individual strains, which is difficult to achieve when applying commercial mixtures of microbes. In any case, no general trend has emerged yet in the effects that an increase of microbial complexity has on the microbe–plant–insect interactions, with evidence showing stronger (Saravanakumar et al., 2007; Currie et al., 2011), weaker (Gange et al., 2003), and no effects (Martinuz et al., 2012) on herbivores aboveground.

One of the factors that can determine the effectiveness of a mixture of microbial strains on plant-mediated effects against herbivores is their genetic relatedness. In a recent study, the effects of four genotypes of the mycorrhizal fungus *R. irregularis*, inoculated alone or in combination, on strawberry plant growth and resistance to the generalist herbivore caterpillar *S. littoralis* were assessed (Roger et al., 2013). Caterpillar fresh weight was reduced by most mycorrhizal treatments, with similar effects of single or dual fungal inoculations. Interestingly, when compared to single inoculation, dual inoculation of genetically very distant isolates affected plant performance parameters stronger than dual inoculation of closely and moderately related isolates. Although in this example herbivore performance was not affected, this could be one of the criteria when searching for powerful combinations of microbes to promote plant growth.

**FIGURE 1:**
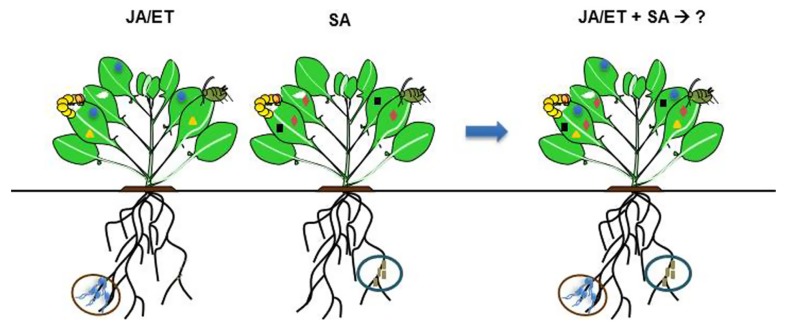
**Selected species of root-associated microbes are known to elicit induced systemic resistance (ISR) by priming for enhanced expression of plant defense-associated genes which become active only after insect or pathogen attack.** Depending on microbe species (indicated in brown or blue circles) or strain, ISR can be triggered via JA/ET- or SA-signaling pathways, in which each pathway activates different sets of defense-associated genes. It is hypothesized that application of multiple root-associated microbes that mediate ISR via different signaling pathways may activate higher diversity of defense-associated genes that can enhance plant defense against insects or pathogens. Crosstalk between multiple signaling pathways (JA/ET–SA) regulating ISR within the plant and on how it will affect the outcome of interactions is not known. Different shape of symbols in the leaves represent different defense- associated genes.

A different factor to consider when combining strains is the change in physiology that the microbial strains induce in the plant. Evidence is accumulating that different strains of root-colonizing microbes can mediate ISR via different signaling pathways (Van Oosten et al., 2008; Van Wees et al., 2008; Jung et al., 2012; van de Mortel et al., 2012). In *A. thaliana*, the strains *P. fluorescens* WCS417r and SS101 decrease the performance of the generalist leaf chewer *Spodoptera exigua* (Van Oosten et al., 2008; Van Wees et al., 2008; Jung et al., 2012; van de Mortel et al., 2012). Whereas strain WCS417r is known to induce resistance to pathogens via JA- and ET-dependent signaling pathways (Pieterse et al., 1998), strain SS101 acts via the SA-pathway and induction of glucosinolate and camalexin biosynthesis (van de Mortel et al., 2012). From these examples, we may speculate that the combined application of root-associated microbes acting via different phytohormonal signaling pathways may enhance plant defense to either pathogens or insect herbivores (**Figure 1**). Supporting this idea, in cucumber, co-inoculation of non-pathogenic *Trichoderma harzianum* and *Pseudomonas* sp. contributed to a significantly enhanced level of resistance upon challenge by the stem pathogen *Fusarium oxysporum* by activating both JA- and SA-dependent defense responses in comparison to individual treatments (Alizadeh et al., 2013). In accordance, the expression of the defense-associated genes *β-1,3-glucanase*, *CHIT1*, *PR1*, encoding glucanase, chitinase, and pathogenesis-related protein respectively, were significantly more pronounced after treatment with a mixture of microbes than with individual strains. Whether activation of both JA- and SA-signaling pathways will also induce the biosynthesis of a higher diversity of secondary metabolites remains to be investigated. Moreover, it can also be hypothesized that some combinations of microbes antagonize each other’s effects due to phytohormonal crosstalk within the plant, but to our knowledge no examples of this have been recorded yet. Investigating the interactive effects of different soil community members is important for a thorough understanding of their plant-mediated effects on insect herbivores.

## PLANT-MEDIATED EFFECTS OF INSECT HERBIVORES ON NON-PATHOGENIC SOIL MICROBES

Upon herbivory, plants respond in several ways that can affect microbe–plant interactions, for instance through the activation of defenses in distal parts, via changes in root exudates, or by modifying soil characteristics. Resistance traits induced in certain plant organs and tissues following pathogen or insect attack can be transported to distant tissues and may affect belowground microbes (Doornbos et al., 2011). For instance, in pepper, sap-sucking whiteflies or aphids induce the up-regulation of both SA-dependent and JA-dependent genes not only in leaves but also in roots (Yang et al., 2011b; Lee et al., 2012b). Interestingly, these defense activations did not equally affect all soil microbes. For instance, repeated leaf mechanical wounding of *Medicago truncatula* increased levels of JA locally and systemically leading to enhanced mycorrhizal colonization, whereas colonization by rhizobacteria was not affected (Landgraf et al., 2012).

Moreover, plants can exude/emit compounds belowground to actively recruit specific belowground beneficial organisms. For instance, attack by the foliar pathogen *Pseudomonas syringae* triggers the secretion of malic acid by *A. thaliana *roots that attract the beneficial rhizobacterium *B. subtilis* (Rudrappa et al., 2008). Foliar infection by the pathogen induced the expression of a malic acid transporter leading to an increased level of malic acid in the rhizosphere (Lakshmanan et al., 2012). Similarly, in maize benzoxazinoids attract *Pseudomonas putida* (Neal et al., 2012). Benzoxazinoids (e.g., DIMBOA) are secondary metabolites that accumulate after herbivory in cereal plants (Erb et al., 2009; Ahmad et al., 2011). Whether they play a role in microbe recruitment after aboveground herbivory remains to be proven. Recently, the first evidence of recruitment of beneficial root microbes after above-ground herbivory has been shown: aphid feeding increased the population of the non-pathogenic rhizobacterium *B. subtilis* GB03 in the rhizosphere of sweet pepper plants (*Capsicum annuum*;**Lee et al., 2012b). However, the chemical cue that triggers the increased colonization has not been discovered yet. This study reveals a new type of interactions and the question arises how multiple herbivory would affect colonization level of root-associated microbes.

Although microbe–plant interactions are established before herbivores will attack those plants, the dynamics of this process are not yet well understood (Heil, 2011). For instance, herbivory may affect via the root exudates certain species of microbes and modify the initial microbiome of a plant. This modified microbiome may have different effects on further herbivore attack on the same plants, or even on the insect interactions with later successional plants. A study using ragwort plants (*Jacobea vulgaris*) showed that both above- and below-ground herbivory gave specific effects on the composition of the soil fungal community, possibly by changing root exudation. Remarkably, these changes affected interactions of preceding plants with aboveground herbivores and parasitoids, providing evidence that herbivory influences plant–soil feedback responses via changes in the community of soil-borne microbes (Kostenko et al., 2012; Bezemer et al., 2013). Evidence that root herbivory influences root-associated microbes via changes in root exudation was also found in maize. Feeding by western corn rootworm (WCR) larvae changes composition of the microbial community in the rhizosphere, depending on soil type and maize line (Dematheis et al., 2012b). This study indicated that the bacterial community was more affected by the presence of WCR larvae than the fungal community. Interestingly, in all soil types an increased abundance of the phenol-degrading bacterium *Acinetobacter calcoaceticus *was found, which was associated with changes in plant root exudation in response to feeding by WCR larvae. Whether changes in microbial communities affect feeding behavior of WCR larvae needs further investigation. Using the same system, they also found the presence of rhizosphere microbes in the gut of WCR larvae. The complexity of the community of rhizosphere microbes in the gut was reduced in comparison with that in the rhizosphere, indicating a highly selective condition of the digestive environment (Dematheis et al., 2012a). The biological role of the rhizosphere-associated microbes in the gut of WCR still needs to be unraveled and can potentially be used in new pest control strategies.

## PLANT-MEDIATED INTERACTIONS BETWEEN ROOT-ASSOCIATED MICROBES AND INSECTS: THE ROLE OF PLANT HORMONES

Plant hormones function as signal molecules regulating plant growth, development and responses to biotic and abiotic stimuli. The phytohormone JA is a lipid-derived compound playing a prominent role in regulating plant growth and defense against various attackers (Browse, 2005; Wasternack, 2007; Pieterse et al., 2012). JA regulates various aspects of plant growth and development such as seed germination, root growth, and flower development (Wasternack, 2007). Moreover, JA functions as the main regulator in the induction of broad-spectrum defense responses to insect herbivores through formation of trichomes as well as enhanced synthesis of proteinase inhibitors (PIs), volatiles, alkaloids, and glucosinolates (Howe and Jander, 2008; Erb et al., 2012). Induction of JA-signaling mainly occurs after attack by necrotrophic pathogens, tissue-chewing insects such as caterpillars, and cell-content feeding insects such as thrips (De Vos et al., 2005).

Jasmonic acid is also responsible for the delivery of long-distance signaling molecules in several plant species (Schilmiller and Howe, 2005; Heil and Ton, 2008; Sogabe et al., 2011; Ankala et al., 2013). Interestingly, JA-signaling has also been described as the main pathway in ISR against aboveground herbivores and is stimulated by root-associated microbes (Van Oosten et al., 2008; Pineda et al., 2010; Jung et al., 2012). The activation of the JA-signaling pathway also affects the plant’s interaction with root-colonizing microbes, for instance by altering the composition of root-associated bacterial communities (Carvalhais et al., 2013). Herbivory by *Pieris rapae* and *Helicoverpa armigera* caterpillars activated a branch of the JA-signaling pathway that is regulated by the transcription factor MYC2 (De Vos et al., 2005; Dombrecht et al., 2007; Verhage et al., 2011). MYC2 positively regulates the biosynthesis of flavonoids (Dombrecht et al., 2007), widely distributed plant secondary metabolites that often function as feeding deterrents to herbivores and as pigments attracting pollinators (Schoonhoven et al., 2005). Interestingly, flavonoids are also present in root exudates and are crucial in the establishment of rhizobacterial colonization (Ferguson and Mathesius, 2003; Steinkellner et al., 2007; Dennis et al., 2010; Zamioudis and Pieterse, 2012). It is known that exogenous application of the volatile JA-derivative methyl jasmonate (MeJA) increases the release of flavonoids from plant roots (Badri et al., 2008; Faure et al., 2009; Buer et al., 2010). Whether JA-induced synthesis of flavonoids is involved in active recruitment or changes of non-pathogenic soil-borne microbe populations following herbivory remains to be investigated.

In addition to JA, SA is another key hormone regulating plant defense against biotrophic pathogens and against insect herbivores with a piercing-sucking feeding mode, such as aphids and whiteflies (Mewis et al., 2005; Pieterse and Dicke, 2007; Zarate et al., 2007; Kusnierczyk et al., 2008; Wu and Baldwin, 2010). More recent findings also suggest a role of SA-dependent signaling in the plant response to insect herbivore oviposition (Browse, 2009; Reymond, 2013). To activate a defense response, SA signaling is transduced via the regulatory protein non-expressor of pathogenesis-related genes1 (NPR1), which functions as transcriptional co-activator of SA-responsive genes such as pathogenesis-related proteins (*PR*) (Dong, 2004). NPR1 is required to mount ISR against pathogens by different beneficial microbes, independently of the pathways that mediate the ISR (Pieterse et al., 1998; Segarra et al., 2009; van de Mortel et al., 2012) and it also functions as an important node modulating SA- and JA-signaling crosstalk (Spoel et al., 2003; Pieterse and Van Loon, 2004; Pieterse et al., 2012). Concurrently, SA-dependent signaling is crucial in interactions of plant roots with non-pathogenic microbes. It has been suggested that in the initial stage of symbiosis, non-pathogenic microbes are sensitive to SA-regulated defense responses (Zamioudis and Pieterse, 2012). SA-signaling has been reported to negatively affect rhizobial, mycorrhizal, and rhizobacterial colonization (van Spronsen et al., 2003; Doornbos et al., 2011). In plant–rhizobia interactions, transient overexpression of *NPR1* in *M. truncatula* suppressed symbiosis, whereas inhibition of *NPR1* induces the acceleration of *Sinorhizobium meliloti* symbiosis (Peleg-Grossman et al., 2009). This suggests that initially the plant recognizes non-pathogenic microbes as alien organisms and, therefore, activates defense mechanisms via SA-dependent signaling pathways (Zamioudis and Pieterse, 2012). In the context of multiple herbivore attack, how crosstalk between signaling pathways induced by insects with different feeding characteristics will affect the level of colonization by root-associated microbes is an area for future investigation.

The JA-signaling pathway also cross-communicates with the ET and ABA signaling pathways through the use of common transcription factors. In *A. thaliana*, the JA-pathway has two main branches, the MYC2- and ERF-branches, each activating different sets of JA-responsive genes (Lorenzo and Solano, 2005; Wasternack, 2007; Pieterse et al., 2012; Kazan and Manners, 2013). The MYC2-branch acts in synergy with ABA-signaling, whereas the ERF branch cross-communicates with the ET-signaling pathway (Abe, 2002; Lorenzo et al., 2003). Herbivory by *P. rapae* and *H. armigera* caterpillars activates the branch that is regulated by the transcription factor MYC2 and enhances the expression of vegetative storage protein 2 (*VSP2*) (De Vos et al., 2005; Dombrecht et al., 2007; Verhage et al., 2011), which is an acid phosphatase having anti-insect activity (Liu et al., 2005). The transcription factor MYC2 is also required to mount ISR against pathogens (Pozo et al., 2008). Recent evidence showed the importance of ABA and ET signaling also in the colonization of plants by non-pathogenic microbes (Camehl et al., 2010; Martin-Rodriguez et al., 2011). In *Arabidopsis*, overexpression of ERF1 had a strong negative effect on root colonization by the beneficial fungus *Piriformospora indica *(Camehl et al., 2010). This study suggested that ET-signaling and ET-targeted transcription factors are crucial to balance beneficial and non-beneficial traits in the symbiosis. In tomato, a functional ABA-signaling pathway was demonstrated to be required for mycorrhization (Martin-Rodriguez et al., 2011). Moreover, there is also negative crosstalk between the ABA- and ET-signaling pathways, in which ABA deficiency enhances the ET level and negatively regulates colonization by mycorrhizae. However, how crosstalk between JA–ABA, JA–ET, and ABA–ET will affect microbe–plant–insect interactions remains to be elucidated.

## POTENTIAL ROLE OF NEW HORMONAL PLAYERS IN REGULATING MICROBE–PLANT–INSECT INTERACTIONS

Increasing evidence shows that the final outcome of plant defense against various attackers is also depending on hormones other than JA and SA (Robert-Seilaniantz et al., 2011). Attention is now shifting to explore plant hormones such as auxin, CK, GA, brassinosteroid (BR), and strigolactone (SL), all of them important in many aspects of plant growth and development (Ohnishi et al., 2006; Sakakibara, 2006; Giron et al., 2013; Liu et al., 2013). For instance, in addition to controlling plant growth via degradation of growth-repressing DELLA proteins, GAs have been indicated to enhance SA-signaling and to increase resistance to biotrophic pathogens (Navarro et al., 2008). Although information on the effect on insect herbivores is scarce (Yang et al., 2011a), the fact that several of these hormones can modulate JA- and SA-signaling (Campos et al., 2009; Ballaré, 2011) suggests that they are also involved in defense responses to herbivores. Interestingly, these hormones are also involved in regulating plant interactions with non-pathogenic microbes. For instance, GA positively regulates nodulation by rhizobia (Ryu et al., 2012), reduced CK levels seem to stimulate mycorrhizal hyphal growth in the roots (Cosme and Wurst, 2013), and SL induces hyphal branching and further establishment of mycorrhizal symbioses (Liu et al., 2013).

Recent experimental evidence suggests that non-pathogenic microbes are able to modify plant hormone metabolism to increase plant growth capacity. In *A. thaliana*, auxin-, BR-, GA-, SA-, and ET-signal transduction pathways are involved in elicitation of growth promotion by several species of non-pathogenic microbes (Ryu et al., 2005; Contreras-Cornejo et al., 2009; Zamioudis et al., 2013). Auxin signaling, known to be critical in regulating plant growth and development, seems to be involved in the effects that non-pathogenic microbes have on root architecture and plant growth (Contreras-Cornejo et al., 2009; Zamioudis et al., 2013). For instance, growth promotion and root development induced by *Trichoderma virens* is reduced in *Arabidopsis*-mutants, *aux1*, *eir1-1*, and *axr1-3*, impaired in auxin-signaling**(Contreras-Cornejo et al., 2009). Several species of non-pathogenic root-associated microbes are known to induce higher auxin concentration *in planta* (Dodd et al., 2010), whereas in response to herbivory, endogenous auxin concentration varies depending on insect feeding mode (Tooker and De Moraes, 2011; Soler et al., 2013). Similarly, intact CK-signaling is responsible for plant-growth promotion by *Bacillus megaterium* in *A. thaliana* (Ortéz-Castro et al., 2008). In lettuce, increased CK content in roots and shoots was observed following colonization of roots by *B. subtilis* (Arkhipova et al., 2005). In plant–insect interactions, CK-related transcripts are strongly upregulated following treatment with fatty acid-amino acid conjugates (FACs), that are insect-derived elicitors (Erb et al., 2012). A very interesting aspect of these hormones for below–aboveground interactions is their role as long-distance signaling molecules (Soler et al., 2013). Auxin has a role in communicating nitrogen shortage between shoot and root (Tamaki and Mercier, 2007). In contrast, CK has been proposed as negative regulator of nitrogen-uptake related genes, which means that CK is produced if an adequate nitrogen level is present, possibly to inhibit nitrogen uptake in the roots (Sakakibara, 2006; Kudo et al., 2010; Kiba et al., 2011). However, how possible crosstalk between JA, SA, and these new hormonal players affects interactions involving microbes, plants, and insects is not known yet.

**FIGURE 2:**
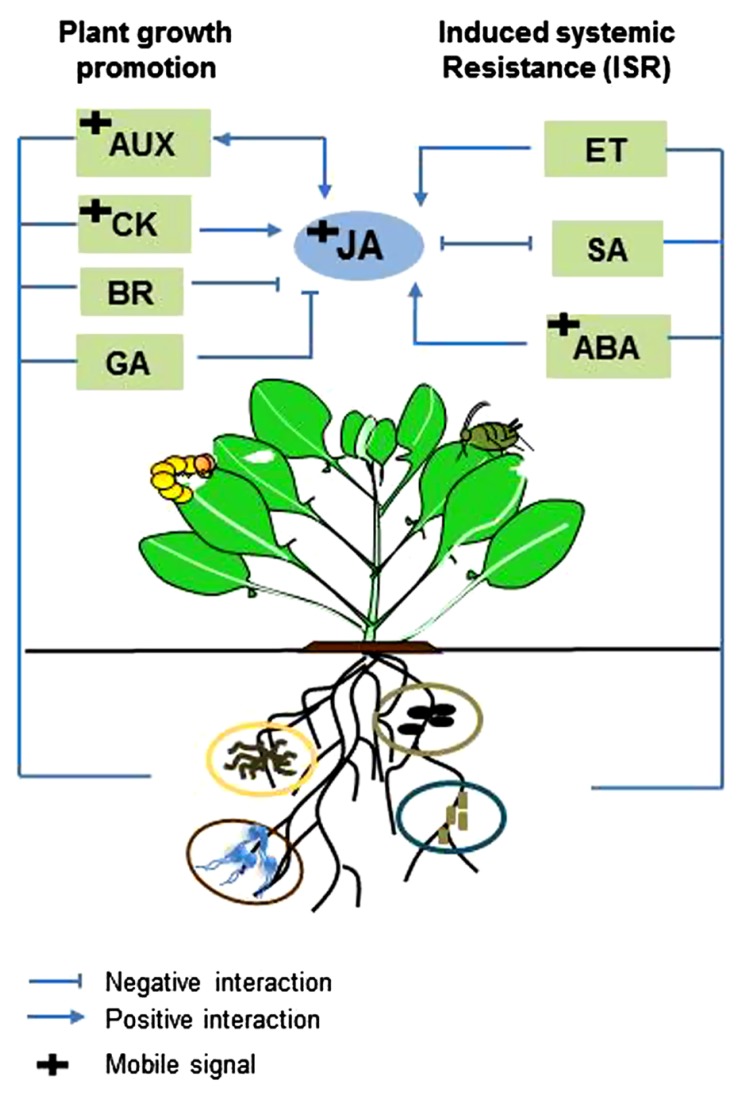
**Model of interactions between plant hormones regulating plant defense and development in microbe–plant–insect interactions.** Different root-associated microbes elicit induced systemic resistance (ISR) via jasmonic acid- (JA), ethylene- (ET,) salicylic acid- (SA) or abscisic acid- (ABA) signaling pathways. Root-associated microbes are also known to induce plant growth promotion via auxin- (AUX), cytokinin- (CK), brassinosteroid- (BR) and gibberellin- (GA) signaling pathways. JA is considered the main hormone regulating the switch from growth to defense through positive and negative crosstalk with other plant hormones. Since root-associated microbes enhance plant defense and growth, these microbes may benefit plant fitness by relieving the trade-off between growth and defense. Investigating the regulatory mechanisms of crosstalk between defense signaling pathways (JA, SA, ET) and growth signaling pathways (AUX, CK, BR, GA) in this system may unveil how plants regulate their resources to invest in growth and defense in the presence of root-associated microbes.

Plants need to regulate resources in the most efficient way to optimally invest in growth and defense. Recent discoveries in plant genomics have shown that hormone signaling networks involved in growth and defense are interconnected, allowing plants to invest in growth under suitable conditions or in defense when they sense attacker-derived signals (Pieterse et al., 2012; Kazan and Manners, 2013). JA has been indicated as the core phytohormone mediating the switch from growth to defense via its positive and antagonistic crosstalk with other plant hormones, such as auxin, GA, and CK (Wasternack, 2007; Pauwels et al., 2009; Ballaré, 2011; Yang et al., 2012; Hou et al., 2013; Kazan and Manners, 2013). In parallel, root-associated microbes are known to increase plant defense and promote plant growth. It is hypothesized that root-inhabiting beneficial microbes can benefit plant fitness by relieving the trade-off between growth and defense (Bennett et al., 2006). However, knowledge on how plants differentially regulate their resources to invest in growth and defense in the presence of beneficial root-inhabiting microbes is not available. Because there is an overlap in how new hormonal players regulate plant defense to insect herbivory and how root-associated microbes promote plant growth, unveiling the regulatory mechanisms of crosstalk between defense signaling pathways (JA, SA, ET) and growth signaling pathways (auxin, GA, CK) and how this will affect the trade-off between growth and defense will be fruitful areas of further investigation (**Figure 2**).

## FUTURE PERSPECTIVES

Over the last two decades, multiple studies in different ecological settings have shown that in nature root-associated microbes can affect insects aboveground. However, the underlying mechanisms of microbe–plant–insect interactions have only recently started to be understood. Using simplified systems with one species of microbe and one species of herbivore, experimental evidence has shown that selected species of root-colonizing microbes may augment plant defense by priming for enhanced expression of defense-associated genes regulated by either JA/ET- or SA-signaling pathways. However, how complex communities of root-inhabiting microbes differentially modulate plant defense and how this will affect herbivores above- and below-ground is a challenging area of future studies. Examples given in this review demonstrate that the application of multiple root-associated microbes can have neutral or even positive effects on the performance of insect herbivores. The fact that in realistic field situations, the positive effect on herbivores could revert to negative effects through increased indirect plant defense by increased attraction of natural enemies indicates the significance of an holistic approach in the study of microbe–plant–insect interactions. Major issues are to gain mechanistic insight in how crosstalk between different microbe-activated signaling pathways affects the level of plant resistance to various insects and to extend studies to natural conditions to assess its ecological implications. Moreover, in response to attack by multiple insect herbivores, plants also activate different hormone-mediated signaling pathways depending on feeding characteristics of the insects and crosstalk between these pathways can have consequences on interactions of plants with root-associated microbes. To our knowledge, no study has addressed how for instance JA–SA crosstalk induced by multiple herbivores would affect the level of mutualistic interactions between plants and root-associated microbes.

Plants have several layers of defense mechanisms to withstand insect attack. In addition to plant indirect defense by attraction of the herbivore’s natural enemies following herbivory, a growing body of evidence shows that to strengthen their layers of defense, plants can actively recruit help from below-ground organisms following attack by foliar pathogens. However, experimental evidence showing that plants develop similar mechanisms following insect herbivory is lacking. If similar mechanisms are uncovered, our understanding of plant defense will grow. Apart from HIPVs emitted by plants, numerous root-associated microbes are also known to produce VOCs that could affect insects directly but it is unknown if microbe-derived VOCs directly influence plant-associated insect communities. The review by Kupferschmied et al., 2013 in this issue provides valuable information on how various traits of root-associated *Pseudomonas* can have direct effects on below-ground pest insects. This hold promise for broader application of root-associated microbes in pest control above- and below-ground.

In their struggle to survive, plants face the dilemma of allocating resources to growth or defense. It is hypothesized that support from root-inhabiting microbes may relieve plants from this trade-off by increasing their access to nutrients (Bennett et al., 2006). However, how plants differentially regulate their resources in the presence of root-associated microbes and which regulatory mechanisms are involved (i.e., hormones, transcription factors) and how these will affect plant interactions with insects still need to be investigated. For instance, crosstalk between JA and SA and between JA and ET in signaling networks is known to be important in the regulation of plant defense against pathogens and insect herbivores. In addition, crosstalk between JA and auxin, JA and GA and JA and CK is thought to play a role in the trade-off between growth and defense. Therefore, it would be interesting to study crosstalk between defense signaling pathways (JA, SA, ET) and growth signaling pathways (auxin, GA, CK) in the context of microbe–plant–insect interactions. Interestingly, several plant hormones that are known to mediate microbe-induced plant growth promotion such as auxin and CK have also recently been identified as mobile signals connecting shoot and roots (Sakakibara, 2006; Tamaki and Mercier, 2007; Kudo et al., 2010). The role of these mobile signals in microbe–plant–insect interactions would be a promising area of further studies.

Beneficial root-associated microbes have a vast potential as environmentally safe pest control agents above- and below-ground. Application of certain species or strains of non-pathogenic bacterial/fungal species into agricultural soils in order to stimulate plant growth or as biocontrol agent against plant pathogens or insects has been performed for years. In spite of several success stories in the application of these non-pathogenic microbes to promote plant health and growth, inconsistencies have often been reported. One of key factors responsible for the failures is the fast decline in the number of microbial populations being introduced, as reviewed in van Veen et al., 1997. The importance of factors such as physiological traits of the microbial agents affecting their competitiveness and survival in the rhizosphere has not been studied in any detail. Interestingly, experimental evidence has shown that specificity of interactions between plant species and associated rhizobacterial communities exist (Smalla et al., 2001; Garbeva et al., 2004; Sugiyama et al., 2013). For decades, application in integrated pest management (IPM) of microbes from the genera *Pseudomonas*, *Bacillus*, and *Trichoderma*, known to colonize many plants from different families, has been common practice. However, the fact that there is a certain level of specificity in the interactions between plant species and their root-associated microbes may indicate that application of certain microbial genera to non-host plants can affect their survival in the rhizosphere. Therefore, we should start identifying plant family-specific groups of root-inhabiting microbes and apply them to their proper host plants to increase their survival in the rhizosphere. Identifying microbial strains from extreme environments, such as insect/disease suppressive soils or the rhizosphere of plants that produce high toxin levels may be a way of obtaining highly competitive microbes. Following the isolation, the characteristics of the isolated microbes in triggering ISR, stimulating plant growth, and competitiveness in the rhizosphere should be evaluated. For a community approach, a thorough selection procedure combining several species of microbes, that are genetically distant and that mediate ISR via different pathways or with different microbe-associated molecular pattern (MAMPs) may enhance competitiveness of the microbes in the rhizosphere, and the induction of ISR. Moreover, understanding the mechanisms and ecology of indirect and direct plant-mediated mechanisms operating between communities of root-associated microbes and insect communities above- and below-ground can increase the reliability and durability of application of beneficial microbes in IPM.

## Conflict of Interest Statement

The authors declare that the research was conducted in the absence of any commercial or financial relationships that could be construed as a potential conflict of interest.
